# Neuromodulation Strategies in Post-Traumatic Stress Disorder: From Preclinical Models to Clinical Applications

**DOI:** 10.3390/brainsci9020045

**Published:** 2019-02-19

**Authors:** Flavia V. Gouveia, Darryl C. Gidyk, Peter Giacobbe, Enoch Ng, Ying Meng, Benjamin Davidson, Agessandro Abrahao, Nir Lipsman, Clement Hamani

**Affiliations:** 1 Sunnybrook Research Institute, Toronto, ON M4N3M5, Canada; flavia.venetuccigouveia@sunnybrook.ca (F.V.G.); darryl.gidyk@sri.utoronto.ca (D.C.G.); peter.giacobbe@sunnybrook.ca (P.G.); Nir.Lipsman@sunnybrook.ca (N.L.); 2 Harquail Centre for Neuromodulation, Sunnybrook Health Sciences Centre, Toronto, ON M4N 3M5, Canada; ying.meng@mail.utoronto.ca (Y.M.); benjamin.davidson@mail.utoronto.ca (B.D.); agessandro.abrahao@sunnybrook.ca (A.A.); 3 Department of Psychiatry, Sunnybrook Health Sciences Centre, University of Toronto, Toronto, ON M4N 3M5, Canada; enoch.ng@utoronto.ca; 4 Division of Neurosurgery, Sunnybrook Health Sciences Centre, University of Toronto, Toronto, ON M4N 3M5, Canada

**Keywords:** deep brain stimulation, fear extinction, post-traumatic stress disorder, transcranial direct current stimulation, transcranial magnetic stimulation

## Abstract

Post-traumatic stress disorder (PTSD) is an often debilitating disease with a lifetime prevalence rate between 5–8%. In war veterans, these numbers are even higher, reaching approximately 10% to 25%. Although most patients benefit from the use of medications and psychotherapy, approximately 20% to 30% do not have an adequate response to conventional treatments. Neuromodulation strategies have been investigated for various psychiatric disorders with promising results, and may represent an important treatment option for individuals with difficult-to-treat forms of PTSD. We review the relevant neurocircuitry and preclinical stimulation studies in models of fear and anxiety, as well as clinical data on the use of transcranial direct current stimulation (tDCS), repetitive transcranial magnetic stimulation (rTMS), and deep brain stimulation (DBS) for the treatment of PTSD.

## 1. Introduction

Post-traumatic stress disorder (PTSD) is an often devastating disease with prevalence between 5–8% [1,2,3,4]. In war veterans, these numbers are even higher, reaching approximately 10–25% [5,6]. It occurs more frequently when the trauma involves certain features, including serious injury, threat of death, or threat to physical integrity. Other types of trauma associated with the development of PTSD include sexual assault, serious accidents, and the unexpected death of a loved one. The symptoms of PTSD are complex, and involve intrusive thoughts, re-experiencing the events, the persistent avoidance of stimuli associated with the trauma, alterations in mood, and persistent symptoms of hyperarousal. Feelings of fear and helplessness are also associated with PTSD. In addition to suffering, PTSD affects multiple dimensions of the patients’ lives, posing substantial stress to family members and caregivers. Due to loss of productivity and increased health costs, the economic burden associated with PTSD may be substantial. 

The most common treatment strategy for PTSD involves the use of psychotherapy and/or medications. The former includes cognitive behavioral therapy and exposure therapy (e.g., trauma-focused cognitive behavioral therapy, eye movement desensitization and reprocessing, and prolonged exposure) [7,8,9,10,11]. Antidepressants, such as selective serotonin reuptake inhibitors and serotonin-norepinephrine reuptake inhibitors are considered first-line medications [11]. Of all the individuals treated with these regimens, up to 20% will relapse within six months [12]. For these patients, alternative pharmacotherapies include the use of second or third-line agents (e.g., mirtazapine, aripiprazole, carbamazepine) or adjunctive therapy (e.g., olanzapine, risperidone, clonidine) [11]. Ultimately, conventional therapies will be ineffective in 20–30% of patients [13]. Both non-invasive and invasive neuromodulation strategies have been proposed to treat this population, including transcranial direct current electrical stimulation (tDCS), transcranial magnetic stimulation (TMS), and deep brain stimulation (DBS).

In this review, we describe the effects of delivering stimulation to structures involved in the neurocircuitry of fear in preclinical models, as well as clinical studies in which neuromodulation strategies have been applied to treat patients with PTSD.

## 2. Preclinical Models

PTSD has been commonly described as a disorder of dysfunctional fear extinction [14]. Many who experience a traumatic event experience physical symptoms and an acute posttraumatic response in the aftermath [15]. Though hypervigilance, anxiety, and avoidance of trauma-related memoranda are common, most individuals’ symptoms resolve over time. In PTSD, fear may not extinguish, and reminders can elicit dysfunctional responses long after the traumatic event [16,17].

It has been hypothesized that individuals who are predisposed to developing anxiety disorders and trauma-related disorders are more sensitive to aversive stimuli and exhibit exaggerated fear responses compared to controls [18]. Indeed, hypersensitive individuals, such as those diagnosed with PTSD, exhibit greater sympathetic responses (e.g., skin conductance and heart rate changes) to visual and imagined threat stimuli as well as to image-based conditioning [19,20,21]. 

As PTSD is a complex clinical disorder, mimicking all its aspects in preclinical models has been challenging [22,23]. In fact, the majority of the currently proposed animal models are more suited for the study of short-term trauma-induced changes in fear and anxiety [22].

A widely used rodent model involves fear conditioning/extinction. During fear conditioning, the individual learns to associate a neutral conditional stimulus (CS)—e.g., tone—with an aversive event featuring an unconditioned stimulus (US); e.g., foot shock [24]. This results in the development of conditioned fear responses (CRs) such as freezing, indicating a learned CS-US association [25]. Fear extinction involves the gradual attenuation of a previously learned CR by repeated presentations of the CS in the absence of US. Rather than deleting previously learned memories, the individual creates a new memory that serve to suppress the original fear response [26].

For the study of anxiety-like behaviors in rodent models, two paradigms have been commonly used: defensive burying and an elevated plus-maze [22,23]. Defensive burying tests the animal’s innate response to bury threatening, dangerous, or aversive objects. Treatments that reduce defensive burying are considered to be anxiolytic [22]. The elevated plus-maze consists of a plus-shape apparatus that contains two open and two enclosed arms and is elevated from the floor. Anxiolytic treatments increase the time the animal spends in the open arms [27]. 

## 3. Neurocircuitry

**Figure 1:**
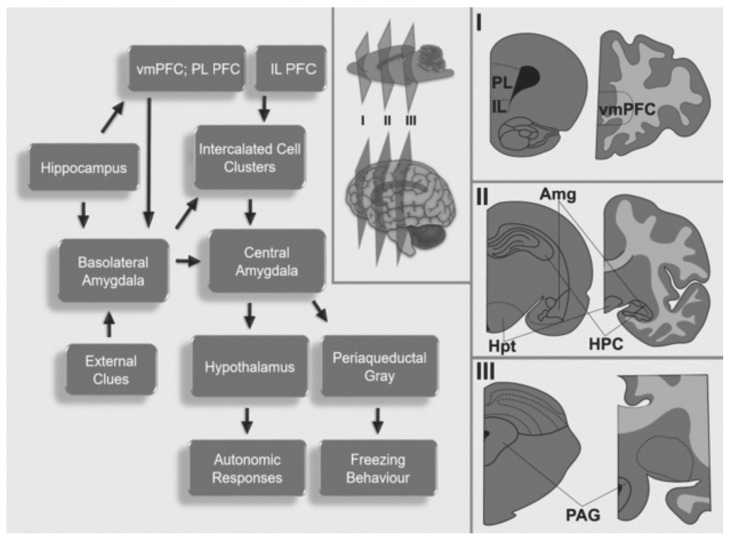
Schematic representation of the neurocircuitry involved in fear conditioning and extinction as well as post-traumatic stress disorder. I, II, and III represent the different coronal sections of rodents (left) and humans (right). Amg, amygdala; HPC, hippocampus; Hpt, hypothalamus; PAG, periaqueductal gray; PFC. Prefrontal cortex; vmPFC, ventromedial prefrontal cortex; IL, infralimbic cortex; PL, prelimbic cortex.

PTSD is a complex disorder comprised of aversive emotional learning (from one or more traumatic events) and dysfunctional memory processes that trigger fear responses without the presence of the traumatic situation [28]. Brain regions associated with PTSD are the amygdala (AMG), hippocampal formation (HPC), and prefrontal cortex (PFC) [29]. Figure 1 shows the main neurocircuitry involved in PTSD and fear conditioning/extinction. 

The amygdala is an oval structure located in the temporal lobes. It is composed of distinct nuclei with particular connections and functions [30]. The amygdala may be subdivided into the central (CE) and medial nuclei (ME), intercalated cell clusters (ITC), and the basolateral amygdalar complex (BLA), which includes the lateral nucleus (LA), basal nucleus (BA), and the accessory basal nucleus [30].

The BLA receives extensive projections from subcortical [31] and primary neocortical auditory, visual, and somatosensory regions [32]. This nuclear complex is responsible for the integration of external and internal cues; thus, it is involved in the amalgamation of aversive events (US) with a non-threatening situation (CS) [33]. Information from the BLA propagates to the CE, and from there to the hypothalamus and multiple brainstem areas, including the periaqueductal grey (PAG) [34]. The PAG is involved in the expression of motor behaviors, such as freezing [35]. The hypothalamus engages autonomic responses and modulates the activity of the hypothalamic-pituitary adrenal axis [36].

Adjacent to the amygdala lies the hippocampal formation (HPC), which is a complex structure formed by the hippocampus (cornu ammonis, CA, dentate gyrus, DG) and subiculum [37,38]. The primate hippocampal formation is subdivided into anterior (ventral in rodents) and posterior (dorsal in rodents) regions [39].

The HPC is not always required for the acquisition, maintenance, and recall of conditioned fear memories. However, it plays an important role in discriminating context-dependent memories (e.g., spatiotemporal/configured representations) to restrain the overgeneralization of fear memories. In PTSD, fear responses are overgeneralized, and occur in non-threatening contexts that are similar to the traumatic experience [28,40].

The PFC can be divided into the ventromedial prefrontal cortex (vmPFC) and dorsolateral prefrontal cortex (dlPFC). The vmPFC is responsible for the integration of sensorial, emotional, environmental, and memory cues, whereas the dlPFC controls the expression of appropriate behavioral responses [41]. In PTSD, the vmPFC appears to be hypoactive, whereas the amygdala exhibits increased activity [42]. Neuroimaging studies in military veterans [43] and sexually abused women [44] have shown decreased blood flow in the vmPFC during the presentation of trauma-related imagery, sounds, or narratives. Reduced vmPFC blood flow has also been reported in PTSD patients during emotional processing tasks [45], after trauma-reminding cues [46], and when at rest [47]. As for the hippocampus, neuroimaging data in PTSD patients have been less consistent. While some studies have shown hypoactivity of the HPC during specific trauma-related tasks [48], others have found increased activity in the left HPC during the encoding and recognition of emotional words in a declarative memory task [49]. It is believed that the dlPFC is responsible for the control and regulation of the vmPFC-AMG-HPC network activity, and that modulating the dlPFC activity may be beneficial in ameliorating key PTSD symptoms [29].

In rodents, rostral cortical regions include the medial prefrontal cortex (mPFC) and the orbital frontal cortex (OFC). Subregions of the mPFC include the anterior cingulate cortex (ACC), the prelimbic cortex (PL), and the infralimbic cortex (IL) [50,51]. While the IL is important for inhibiting fear CRs following extinction learning [52,53], the PL is primarily involved in the long-term behavioral expression of fear [53,54]. The IL appears to modulate the inhibition of fear responses by activating GABAergic ITC [54], which also receive excitatory input from the BLA, and send inhibitory efferents to the CE [55]. During normal extinction learning, IL inputs activate the ITC, thus inhibiting the CE and reducing overt fear responses (i.e., freezing) [56].

## 4. Stimulation in Preclinical Models

In rodent models, electrical stimulation has been delivered to various brain structures either to study its behavioral consequences or understand the role of specific regions in mechanisms of fear conditioning/extinction and anxiety. Overall, electrical stimulation through implanted electrodes (analogous to DBS) is an invasive technique that acts through complex mechanisms. Briefly, a single pulse or trains of pulses delivered at low frequencies result in the excitation of axonal pathways and cell depolarization. However, though stimulation delivered at higher frequencies (e.g., >90 or 100Hz) is still able to activate fibers, it leads to the functional inactivation of cell bodies (e.g. depolarization block) [57,58]. Under these circumstances, stimulation may modulate the activity of structures at a distance (i.e., though the activation of axonal projections), while inhibiting firing in the target region [22,59]. DBS has also been shown to modulate oscillatory brain activity, neurotransmitter release, and induce several forms of plasticity. 

As introduced above, fear extinction involves the creation of new memories that permit the suppression of original fear responses (CR; e.g., freezing). Thus, memory for the conditioning stimulus still exists after extinction, but the fear response previously evoked by CS becomes suppressed [26]. In PTSD, there is a diminished executive suppression of the HPC-AMG-vmPFC neurocircuitry, resulting in the overgeneralization of fear memories and persistent fear responses [28]. 

Low-frequency simulation of the ventral CA1 region of the hippocampus following contextual conditioning reduced freezing during extinction [60], whereas stimulation of the dorsal CA1 or intermediate CA2 following extinction reduced plasticity and increased freezing during extinction recall tests [61,62]. Inversely, high-frequency stimulation delivered to the HPC after fear conditioning/extinction has been shown to enhance extinction and reduce freezing during recall sessions [63].

High-frequency stimulation of the ventral striatum was tested in a fear conditioning and extinction paradigm. While animals with electrodes implanted dorsal to the anterior commissure had significantly less freezing than sham controls, those implanted with ventral electrodes had the opposite response [64]. Similarly, high-frequency stimulation of the BLA was shown to reduce tone, but not contextual fear memory following conditioning [65]. Furthermore, BLA high-frequency stimulation led to anxiolytic effects in the defensive burying test and elevated plus-maze [66,67].

Several studies applied electrical stimulation to investigate the contribution of the PFC to fear conditioning and extinction. While PL stimulation time-locked to CS impaired extinction, IL stimulation had the opposite effect [52,68]. In addition, IL stimulation delivered at high frequencies for 10 minutes after various stages of conditioning and extinction reduced freezing when animals were re-exposed to the conditioning context [69,70,71]. In a recent study, we found that vmPFC stimulation delivered chronically (e.g., two weeks) to rodents with high levels of freezing following extinction reduced this behavioral response and improved anxiety [72].

Also tested in rodent models were rTMS and tDCS. These are non-invasive neuromodulation techniques that alter local electrical activity, neuronal firing, and may sometimes lead to changes at the circuitry level [29,73]. TMS uses focused electromagnetic pulses (trains of stimulation) and different simulation parameters to increase (high-frequency stimulation: > 1 Hz) or decrease (low-frequency stimulation: ≤ 1 Hz) cortical excitability [74]. tDCS uses constant direct current to increase or decrease cortical excitability by depolarizing (anodal tDCS) or hyperpolarizing neurons (cathodal tDCS) [74]. 

In a fear condition/extinction paradigm, rTMS delivered during extinction resulted in a more pronounced reduction of freezing when compared to sham stimulation [75]. It also reduced anxiety-like behavior in the elevated plus-maze and plasmatic corticosterone levels [76]. When assessing the biological mechanisms of this technique, high-frequency rTMS was shown to increase immediate early gene expression in the PFC (infralimbic region), BLA, and ventral CA1 [77], enhance glutamatergic transmission in the ACC, and influence the PTEN/Akt signaling pathway, which is involved in the regulation of memory and synaptic plasticity [78].

The use of stimulation procedures analogous to those used in humans is crucial for a better appraisal of how neuromodulation techniques restore normal fear responses. However, these are unsuited to identifying the cellular and physiological mechanisms of fear and anxiety. Optogenetics is a unique technique that permits the evaluation and manipulation of discrete aspects of physiological and disease states [79,80]. This is in contrast to the multiple changes and numerous substrates affected by clinical neuromodulation strategies. Work on the use of optogenetics to dissect mechanisms of fear and anxiety is quite extensive, and has been reviewed elsewhere [81,82,83,84]. 

## 5. Neuromodulation Studies in Clinical Practice

In clinical practice, neuromodulation treatments are being investigated for the amelioration of PTSD in patients who are resistant to conventional therapy. The non-invasive characteristic of rTMS and tDCS has placed them in the spotlight [85]. 

**Figure 2:**
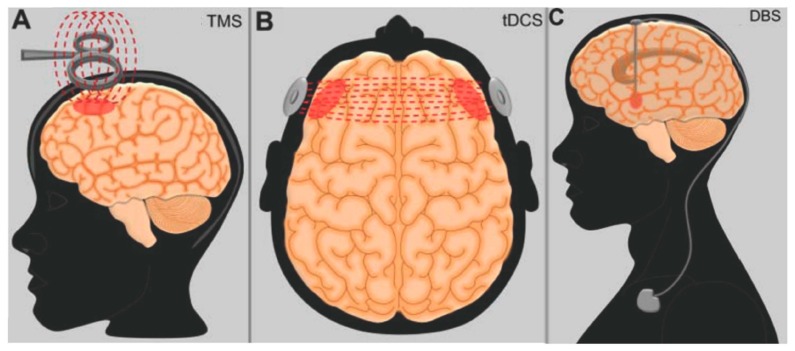
Schematic representation of neuromodulation treatments investigated in patients with post-traumatic stress disorder. (**A**) Transcranial magnetic stimulation (TMS); (**B**) transcranial direct current stimulation (tDCS); (**C**) deep brain stimulation (DBS).

As described above, the overgeneralization of fear memories and the persistent expression of fear responses are consequences of a dysregulated neurocircuitry that mainly includes the amygdala, prefrontal regions, and hippocampus. It is believed that a treatment that is capable of augmenting the activity of prefrontal regions could be effective in ameliorating the executive control of fear responses, thus improving PTSD [73,74]. Along this line, researchers are investigating the use of rTMS and tDCS in the region of the PFC (Figure 2A,B).

Patients who have been included in rTMS studies were mostly veterans with comorbid depressive symptoms resistant to conventional treatments. The typical locus of stimulation was the dorsolateral prefrontal cortex, either unilaterally (right or left) or bilaterally. Stimulation frequencies have largely varied from 1 to 20 Hz and motor thresholds (MT) have varied from 80% to 120% [29,86,87,88,89,90,91,92,93,94]. All studies but one [90] have reported an improvement of PTSD symptoms (i.e., measured with the PTSD Checklist-Military Version, PCL-M; PTSD Checklist-Civilian Version, PCL-C; PTSD Checklist for DSM-5; or Clinician Administered PTSD Scale, CAPS). One episode featuring a severe adverse event (tonic-clonic generalized seizure) and a few mild adverse events (headache, dizziness) have been reported [29,87,88,89,90,91,92,93,94].

A double-blind, placebo-controlled phase II trial was performed to study the efficacy of unilateral dlPFC rTMS in ameliorating PTSD symptoms (20 Hz, two seconds of every 30 seconds, MT 80%) [88]. Ten patients received sham, and 10 received active stimulation. Although improvement was observed in both groups, patients receiving rTMS had a greater amelioration of symptoms [88]. Another randomized clinical trial investigated rTMS (1 Hz, MT 110%) applied to the right dlPFC prior to cognitive processing therapy for the treatment of combat-related PTSD. A greater improvement across sessions was observed in the active rTMS group, in which benefits were sustained for up to six months [87]. In contrast to these findings, a recent randomized controlled trial found no difference in remission rates when patients receiving active versus sham TMS were compared [95]. In that study, patients were randomized to receive left prefrontal rTMS (10 Hz, 120% motor threshold) or sham treatment. Of the 164 patients included, 81 received active left prefrontal rTMS, and 83 received sham rTMS. A primary analysis of remission (Hamilton Rating Scale for Depression score ≤ 10) revealed no significant differences between groups (40.7% in the active group, and 37.4% in sham-treated patients). PTSD symptoms in both groups were also similar [95].

tDCS studies in PTSD have largely focused on delivering stimulation along with psychotherapy, in order to potentially boost the effects of psychological interventions. A pilot study that associated prefrontal cortex tDCS (anode AF3, cathode M1) with computerized memory training showed improvements in working memory and PTSD symptoms in four veterans [96]. In a different study, the effects of tDCS (anode AF3, cathode P08) on extinction learning and consolidation were tested in 28 veterans after fear conditioning [80]. Using skin conductance as the dependent measure, results indicated that tDCS improved extinction recall and enhanced the efficacy of psychotherapy. The only randomized controlled trial using tDCS for PTSD was designed to test whether tDCS could improve extinction recall. Twelve veterans were evaluated in a virtual reality paradigm (combat-related) associated with tDCS (anode over AF3; cathode over PO8) or sham stimulation. Patients were stable on other treatments prior to and during the study. Using normalized skin conductance reactivity and the PTSD Checklist for DSM-5 (PCL-5) scale as the main outcome measures, the authors found a significant reduction in arousal and the severity of PTSD symptoms [97]. 

In contrast to non-invasive modalities, DBS involves the administration of electrical current through electrodes implanted in the brain parenchyma (Figure 2C). Although a surgical procedure is required for system implantation, the technique is considered to be safe [98], and its results are often long-lasting [99]. To date, one PTSD patient treated with BLA DBS has been reported [100]. Eight months after surgery, he had an improvement in CAPS scores of approximately 40%. Overall, the procedure was found to be safe, with no seizures or after-discharges being recorded during electrophysiology analyses.

## 6. Conclusions

There are currently no optimal treatment alternatives for patients with PTSD that are deemed to be refractory to medications and psychotherapy. Neuromodulation strategies have been successfully used in depression and obsessive compulsive disorder, and are currently being investigated for this population. With a well-characterized neurocircuitry, research into treatment alternatives for PTSD may lend itself well to direct-to-brain, neuroanatomically-guided neuromodulation strategies. Given the profound burden of this illness, it is anticipated that a higher number of studies will be published in the future evaluating the therapeutic effects of rTMS, tDCS, and DBS in the management of treatment-resistant PTSD.
